# Paroxysmal Supraventricular Tachycardia in Wolff–Parkinson–White Syndrome in a Newborn—Case Report and Mini-Review

**DOI:** 10.3390/medicina56110588

**Published:** 2020-11-05

**Authors:** Alina-Costina Luca, Alexandrina-Stefania Curpan, Ingrith Miron, Emma Oana Horhota, Alin-Constantin Iordache

**Affiliations:** 1 Faculty of Medicine, “Grigore T. Popa”, University of Medicine and Pharmacy, Strada Universitatii 16, 700115 Iasi, Romania; acluca@yahoo.com (A.-C.L.); lucmir@gmail.com (I.M.); emmahorhota@yahoo.com (E.O.H.); aliniordache@yahoo.com (A.-C.I.); 2 Department of Biology, Faculty of Biology, “Alexandru Ioan Cuza” University of Iasi, Bd. Carol I, 20A, 700505 Iasi, Romania

**Keywords:** Wolff–Parkinson–White, arrhythmia, tachycardia, PSVT, associated conditions

## Abstract

Wolff–Parkinson–White (WPW) syndrome is a rare abnormal condition frequently associated with paroxysmal supraventricular tachycardia (PSVT) and is described as an arrhythmia under the form of increased heartbeat. Currently, there are various possible treatments going from medicines such as adenosine and beta-blockers to cardioversion. The unknown causes of this condition together with the different responses to treatment in each patient make it difficult to establish the best therapeutic approach. In this context, in the current paper, we were interested in reporting the therapeutic options and their efficiency in the case of associated heart or inflammatory conditions in a 13-day-old patient.

## 1. Introduction

Wolff–Parkinson–White (WPW) syndrome is an abnormal condition associated with abnormal heartbeat and it is a result of a pathological pathway between the atria and the ventricles that surrounds the electrical conduction pathway of the atrioventricular node. Patients with WPW are frequently susceptible to paroxysmal supraventricular tachycardia (PSVT) [1].

The incidence of this syndrome is 0.9–3% in the general population. In order to validate the WPW syndrome, an electrocardiogram (EKG) with specific parameters (short P–R interval, Δ wave, and wide QRS) needs to be conducted [2]. WPW can often mimic other anomalies, for instance, ventricular hypertrophy [1]. In the case of children younger than 2 months who already had their first episode of WPW supraventricular paroxysmal tachycardia-associated syndrome, they exhibited the loss of tachycardia in 93% of the cases, but it recurred in 31% of the cases after the 8-year-old mark [3].

Supraventricular tachycardia is an arrhythmia described by an increased heartbeat with its origin above the ventricular tissue [1]. It can occur in 15% of the cases as a follow-up of cardiac congenital malformations, medication, acute conditions [4], or other types of inflammation [5], with the most frequent origin being localized on the heart, but without affecting its structure or an already existing cause.

The prevalence of this disease can vary from 1 in 25,000 to 1 in 250 children [4]. From the onset time point of view, it was revealed as being most frequent in children younger than 1 year old with a percentage of 25%; however, multiple studies suggest that PSVT has 2 onset peaks, one during the nursling period and one between the ages of 8–12 years [5,6,7,8,9].

## 2. Case Presentation

Our patient was a 13-day-old female with a past medical history of being an emergency born through C-section during the 37th week of pregnancy due to an elevated heart rate (HR) during echocardiography (250 beats per minute (BPM)) and a systolic murmur grade II out of VI detected at birth, who presented to the emergency department (ED) in a general serious condition characterized by food refusal, jaundice of the skin, cyanosis mucosa, tachycardiac heart noises, HR = 260–280 BPM, subcostal draught, polypnea, depressible abdomen, absent stool, hypotonic, drowsy, and reactive to touch and painful stimuli.

After specialty investigations, the case was treated as a paroxysmal tachycardia in newborns; therefore, the oculocardiac reflex test was performed in the ED with no response leading to adenosine administration (one dose) with reconversion converted to the heart rate sinus rhythm (HR = 140–160 BPM). Subsequently, after an hour and a half, the patient became tachypneic, developed moaning, lethargy, and suprasternal and subcostal indrawing requiring endotracheal intubation and ventilation by means of the intermittent positive-pressure ventilation (IPPV) system (80–95% pre and post ductal SaO_2_ saturation and uneven pre and post ductal HR (preductal 180 BPM, post ductal 65 BPM)). The patient exhibited cardiopulmonary arrest reversible with attempted cardiopulmonary resuscitation (CPR), external cardiac massage, mechanical ventilation, and administration of adrenaline (3 doses of 0.3 mL) resulting in her being transferred to the acute therapy unit of the Saint Mary Children Hospital in Iasi.

**Figure 1:**
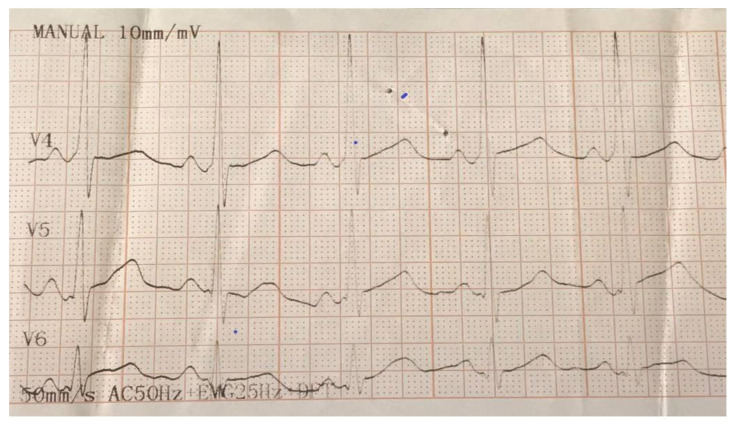

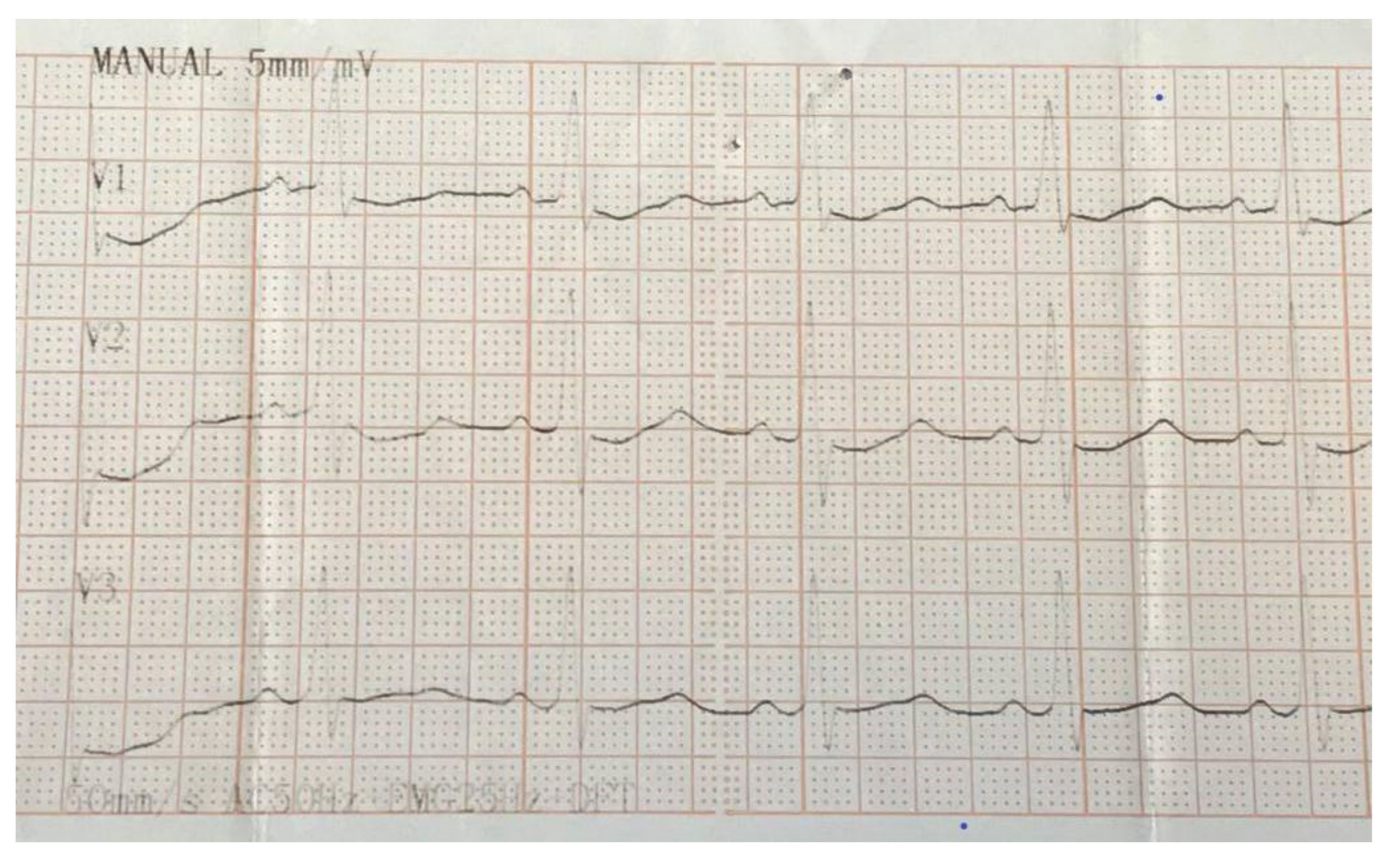
Electrocardiogram (EKG) illustrating delta waves in V1 and V4 as the basis for the diagnosing of Wolff–Parkinson–White (WPW) syndrome.

By using an electrophysiological study, it was possible to diagnose the patient with preexcitation syndrome, lateral left WPW syndrome and electrically reduced orthodromic paroxysmal supraventricular tachycardia. The interventional cardiologist did not consider radiofrequency ablation necessary at that moment, because the evolution was good with medication (the patient did not present other episodes of PSVT) and only recommended adenosine in case other episodes of PSVT occur (Figure 1).

At admission time, her clinical profile included orotracheal intubation and mechanical ventilation, icteric teguments, harsh vesicular murmur, respiratory rate = 54/min, subcostal recession, tachypnea, gallop rhythm, HR = 150–160 BPM, 90% SO_2_, left parasternal systolic murmur II/VI, gavage, depressible abdomen, anterior fontanelle = 2/1 cm, and accentuated pulmonary markings in the hyaline-basal region. The laboratory analysis results included leukocytosis, neutrophilia, monocytosis and normochromic microcytic anemia, thrombocytosis, immunoglobulin G (IgG) and immunoglobulin M (IgM) deficiency, hypoproteinemia, hypocalcemia, and elevated levels of bilirubin. In the tracheal aspirate (collected one day after her transfer to our unit when she was already intubated), we identified *Acinetobacter baumannii*. To the best of our knowledge, the source of infection is unknown (whether she contacted it at birth or during intubation), but we believe that the infection either triggered PSVT or worsened the patient’s condition. Echocardiographic examination detected global heart failure, patent foramen ovale, patent ductus arteriosus, coarctation of the aorta, atrial septal aneurysm, right deviated, partial anomalous pulmonary venous return, grade II mitral regurgitation, and ventricular diastolic dysfunction.

**Figure 2:**
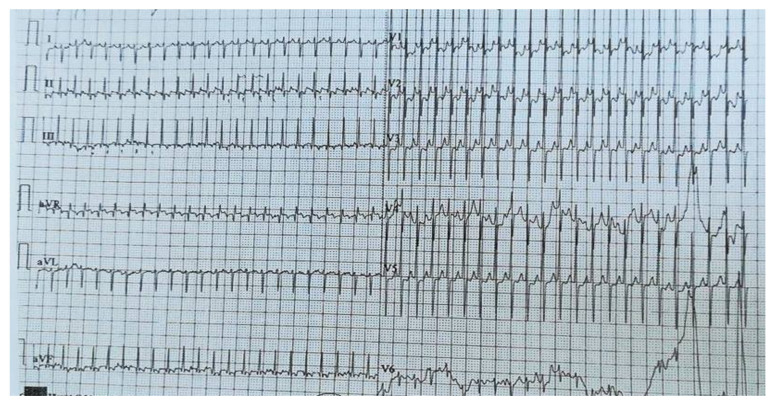
EKG in crisis. Sinus rhythm, heart rate = 270 b/min, supraventricular tachycardia, right axis deviation, right ventricular hypertrophy, and widespread ST–T abnormality.

After 19 h from the first PSVT episode, the patient had a second episode (atrioventricular (AV) = 295 BPM). The therapy, this time, consisted of a dose of adenosine 0.1 mg/kg (0,4 mg), unsuccessful in restoring the sinus rhythm, followed by 2 more doses of the same value, unsuccessful as well (AV = 276 BPM). Thus, the patient received half a dose of digoxin 0.04 mg/kg of body weight (the other half was divided into halves itself, the first being administered at the 8 h mark and the second at the 16 h mark), which led to AV dropping at 67 BPM, requiring the administration of 0.3 mL of intravenous adrenaline. In view of the fact that, two hours later, the AV greatly increased again (270 BPM) (Figure 2) and the liver was palpable at 4 cm under the rim, we chose to associate the therapy with beta-blockers and follow up with amiodarone and dobutamine. None of the therapeutic maneuvers was successful, thus we opted to use cardioversion at 1.25 J/kg (5j) (5 h after the first dose of adenosine was administrated for the second episode), which turned out to be the right choice, as it was able to restore the sinus rhythm (HR = 110 BPM).

The patient did not have another PSVT episode during her hospitalization; she was extubated, and her alimentation switched back to nursing. She continued treatment with ceftazidime, digoxin, calcium gluconate, magnesium, furosemide, spironolactone, Ursofalk, midazolam, 1/3 vial of albumin for 5 consecutive days, and she also received 50 mL of blood transfusion (O type, Rh+). At discharge, we recommended that the patient continue treatment with spironolactone, furosemide, and digoxin for another month and return for reevaluation. At reevaluation, she presented in good general condition, with sinus rhythm AV = 120 BPM and systolic murmur grade II out of VI. To our knowledge, the patient is now 2 years old and is reevaluated by the pediatrician in her region (she is currently in Germany) and has not presented other episodes of PSVT.

## 3. Discussion

PSVT can be diagnosed as early as in uterine life by means of fetal heart rate screening (when heart rate exceeds 180 BPM) and it can be treated through transplacental administration of antiarrhythmic drugs [10]. In children younger than 1 year, HR in PSVT is between 220 and 280 BPM, and in those older than 1 year, it is 180–240 BPM [11]. PSVT can have up to 16 different action mechanisms [11], but the most frequent ones are: through an accessory pathway (73% and the most common in nurslings), through reentering the atrioventricular node (13%) and primary atrial tachycardia (14%) [12], also known as atrioventricular reentrant tachycardia (AVRT), atrioventricular nodal reentry tachycardia (AVNRT), and ectopic atrial tachycardia. In the case of our patient, the diagnosis was confirmed at the 37th week of intranatal life by using perinatal ultrasound. The exact cause is most likely due to associated heart malformations, more exactly issues of embryonic development, with remnant embryonic atrioventricular pathways not undergoing regression. Bassareo and associates (2018) indicated that a key role in triggering PSVT episodes is played by associated inflammatory conditions, a matter difficult to oppose in the case of our patient, which was found with *A.r baumannii* in the tracheal aspirate.

From a clinical point of view, PSVT is difficult to diagnose due to its nonspecific symptomatology (loss of appetite, vomiting, irritability, drowsiness, and diaphoresis) that has the potential to evolve into syncope, paleness, and cyanosis when congestive heart failure is also present [11]. In a study conducted on 27 patients younger than 1 year, and diagnosed with PSVT, 24 were accidentally discovered, while the rest presented symptoms such as paleness, alimentation difficulties, and dyspnea [8]. Moreover, there are situations in which WPW is untraceable and the patients never develop arrhythmia, thus their medical evaluation and even ECG are normal [13].

The emergency treatment for PSVT’s acute stage is first and foremost represented by vagal maneuvers (unilateral carotid sinus massage, applying pressure on the eyes, and applying an ice bag on the child’s face for 10 sec—this is also the most efficient method for nursling). This method was also used for our patient, but with no therapeutic outcome.

In the situation where vagal maneuvers are not effective, the specialty literature advocates for intravenous (IV) or intraosseous (IO) administration of adenosine (starting with a 0.1 mg/kg of body weight dose (6 mg at most)), followed by a saline solution. If the initial dose does not have the desired outcome, a subsequent administration of 0.2–0.3 mg/kg of body weight (maximum 12 mg) is recommended. In our patient, three 0.1-mg/kg of body weight doses were administrated [14]. The study conducted by Losek and associates (1999) on an experimental group of 71 PSVT patients demonstrated a 72% success rate of adenosine. Nevertheless, in only 4 patients, the sinus rhythm was restituted with a dose of 0.1 mg/kg of body weight; in 44 patients, a dose of 0.2 mg/kg of body weight was required, and for 23 patients, a dose of 0.3 mg/kg of body weight was used [6]. Other antiarrhythmic drugs that might be administrated for reducing PSVT are propranolol (in patients with WPW syndrome) as well as other beta-blockers, such as verapamil, digoxin [1], or amiodarone, the efficiency and safety of which was observed in nurslings and may be administrated if adenosine proves to be ineffective [15,16]. Nevertheless, in the case presented by our team, even beta-blockers (metoprolol, clinical use) or amiodarone could not restore the sinus rhythm of the patient. We used digoxin both for antiarrhythmic purposes and for the positive inotropic effect.

In the situation where the therapeutic maneuvers described above are ineffective or when the patient is hemodynamically unstable in cardiogenic shock or congestive heart failure, the patient might be subjected to direct cardioversion from 0.5 J/kg of body weight to up to 2 J/kg of body weight [1,14]. One important aspect that must be considered in this therapeutic option is that an inadequate synchronization to the heart rhythm could lead to ventricular fibrillation or ventricular tachycardia [4]. In our case, direct cardioversion was successful at 1.25 J/kg of body weight, but in the case presented by Dadi, Fink, and Weiser (2017) [17], the cardioversion was unsuccessful even at 2 J/kg of body weight, a situation that made the authors use an increased dose of adenosine (0.4 mg/kg of body weight).

Usually, WPW symptoms disappear in most children by the age of 12 months, but PSVT may reoccur later in childhood as mentioned in the Introduction section. Although it was not the case of our patient, when WPW problems persist and medication does not succeed, or when PSVT occurs in older patients, the effective therapy of choice is ablation. The management of WPW-related PSVT is different due to its high risk of cardiac arrest or sudden death (which is most frequent in people with high-risk occupation and athletes), in which case, electrophysiologic (EP) studies and radiofrequency (RF) catheter ablation may be curative and the treatment of choice. In infants weighing less than 15 kg before performing ablation, there are several factors that need to be considered (which tachycardia is to be ablated, its clinical presentation, alternatives, the potential for success or complications), because they represent a higher risk for complications [18,19].

## 4. Conclusions

In conclusion, every patient will react in a personal and specific manner to antiarrhythmic therapy based on PSVT cause as well as other associated conditions. In our case, the newborn responded to adenosine in the first episode of PSVT, but for the second one, cardioversion was required as no other therapeutic option available in our clinic was able to restore the sinus rhythm.

The therapeutic options are various going from medication to cardioversion and ablation and all possible scenarios have to be analyzed before deciding the route of treatment. Ablation is a highly successful approach with a low complication incidence, but with risks high enough to be carefully analyzed before performing it on pediatric patients, as the benefits of this procedure need to outweigh the risks. As described in this case report, in our 13 day-old patient, treatment with heart rhythm medicine and cardioversion seemed to be effective, but due to the lack of follow-up, it is close to impossible to determine the exact explanation for the clinical improvement.
